# Obsessive-Compulsive Disorder: Neurocognitive Links with Impulsivity and Inhibitory Control

**DOI:** 10.1192/j.eurpsy.2025.2438

**Published:** 2025-08-26

**Authors:** D. V. Cotovio, R. S. Nogueira, R. Lousada, M. B. Resende, F. Agostinho, M. M. Melo, M. J. Heitor

**Affiliations:** 1 Departamento de Psiquiatria e Saúde Mental, Hospital Beatriz Ângelo, Loures, Portugal

## Abstract

**Introduction:**

Obsessive-compulsive disorder (OCD) and attention-deficit/hyperactivity disorder (ADHD) share overlapping neurobiological and neurocognitive profiles, particularly in domains such as impulsivity, compulsivity and inhibitory control. Recent research has explored these commonalities, as well as the differences in brain structure, neural circuits, and cognitive impairments.

**Objectives:**

The aim of this review is to synthesize findings about the neurocognitive and neurobiological profiles of OCD and ADHD.

**Methods:**

A literature search was conducted on PubMed in September 2024 using the following terms: “OCD” AND “ADHD,” “OCD” AND “attention-deficit/hyperactivity disorder,” “obsessive-compulsive disorder” AND “ADHD,” and “obsessive-compulsive disorder” AND “attention-deficit/hyperactivity disorder.” Only systematic reviews and meta-analyses were included, with no year or language restrictions. Seven articles met the scope of this review.

**Results:**

On one hand, the selected studies indicate that both OCD and ADHD exhibit significant deficits in inhibitory control, as measured in the Stop Signal Task and antisaccade task. These findings are corroborated by neuroimaging abnormalities in brain regions implicated in inhibition, that includes the frontostriatal circuits. Studies also suggest that compulsivity may stem from dysfunctional glutamatergic transmission in frontostriatal circuits. On the other hand, though structural analyses reveal a shared pattern of brain alterations in OCD, major depressive disorder, bipolar disorder, and schizophrenia, ADHD appears to have different morphometric properties. Furthermore, electrophysiological studies also demonstrate contrasting patterns of error detection between the disorders: increased error-related negativity (ERN) in OCD and reduced ERN in ADHD.

**Conclusions:**

OCD and ADHD share common neurobiological and neurocognitive traits, yet distinct differences in brain structure and neurocognitive processing also emerge. This review highlights the importance of transdiagnostic approaches, which may facilitate the development of more targeted treatments for patients with overlapping neuropsychiatric conditions. Further research is necessary to understand the full extent of these shared and unique characteristics, in order to inform more refined diagnostic criteria and therapies.

**Disclosure of Interest:**

None Declared

